# Plasticity within the αβ^+^CD4^+^ T-cell lineage: when, how and what for?

**DOI:** 10.1098/rsob.120157

**Published:** 2013-01

**Authors:** Stephanie M. Coomes, Victoria S. Pelly, Mark S. Wilson

**Affiliations:** Division of Molecular Immunology, National Institute for Medical Research, MRC, London NW7 1AA, UK

**Keywords:** T helper cell, T regulatory cell, infection

## Abstract

Following thymic output, αβ^+^CD4^+^ T cells become activated in the periphery when they encounter peptide–major histocompatibility complex. A combination of cytokine and co-stimulatory signals instructs the differentiation of T cells into various lineages and subsequent expansion and contraction during an appropriate and protective immune response. Our understanding of the events leading to T-cell lineage commitment has been dominated by a single fate model describing the commitment of T cells to one of several helper (T_H_), follicular helper (T_FH_) or regulatory (T_REG_) phenotypes. Although a single lineage-committed and dedicated T cell may best execute a single function, the view of a single fate for T cells has recently been challenged. A relatively new paradigm in αβ^+^CD4^+^ T-cell biology indicates that T cells are much more flexible than previously appreciated, with the ability to change between helper phenotypes, between helper and follicular helper, or, most extremely, between helper and regulatory functions. In this review, we comprehensively summarize the recent literature identifying when T_H_ or T_REG_ cell plasticity occurs, provide potential mechanisms of plasticity and ask if T-cell plasticity is beneficial or detrimental to immunity.

## 2. Introduction: T-cell differentiation programmes

The differentiation of αβ^+^CD4^+^ T cells is the result of combined T-cell receptor (TCR) engagement, co-stimulation and distinct cytokine receptor ligation. These three signals, sequential or concurrent, activate and phosphorylate a suite of transcription factors (TFs) that translocate into the nucleus. TFs binding to *cis*-regulatory elements (promoters, enhancers, insulators and silencers) within gene promoter regions translate extracellular signals to downstream transcriptional programmes. Epigenetic changes to *cis*-regulatory elements can influence TF binding and the subsequent fate of the cell, adding a level of regulation at this early stage of cell differentiation. Target gene transcription and translation convert naive T cells into mature T cells with distinguishable features, including the expression of specific adhesion molecules and surface receptors, chemokine-producing capacity and activation of often distinguishable metabolic pathways [1]. Differentiated T helper (T_H_) cells can be defined and distinguished from one another by their primary cytokine-producing capacity, including, but not limited to, interferon (IFN)γ-producing T_H_1 cells, interleukin (IL)-4-producing T_H_2 cells, IL-17A-producing T_H_17 cells and IL-9-secreting T_H_9 cells. Mature T_H_ cells function to mobilize and activate innate cells, re-enforce T_H_ cell commitment and orchestrate local tissue responses through various lymphokine secretions [2]. In addition to a helper fate for T cells, naive αβ^+^CD4^+^ T cells can differentiate into follicular helper T cells (T_FH_) specialized for B-cell help within marginal zones and germinal centres. In contrast, naive αβ^+^CD4^+^ T cells can adopt a regulatory (T_REG_) function with potent suppressive capacities. Several T_REG_ populations have been described, including Foxp3^+^ natural T_REG_ (nT_REG_), which develop in the thymus in response to self-antigen [3], and inducible Foxp3^+^ (iT_REG_) cells, which develop in the periphery in response to exogenous antigen and transforming growth factor (TGF)-β [4]. Non-Foxp3-expressing T_REG_ cells have also been identified, including TGF-β-secreting (T_H_3) [5], IL-10-secreting (T_R_1) [6] or IL-35-secreting (T_R_35) T_REG_ [7] cells; however, in this review, we will focus on Foxp3^+^ T_REG_ cells.

The transcriptional programmes, mediated by a suite of TFs and signal transducer and activator of transcription (STAT) molecules, for the differentiation of T_H_, T_FH_ or T_REG_ cells are mostly well defined. For example, Tbet, STAT-1 and STAT-4 are required for T_H_1 differentiation, GATA-3 and STAT-5 for T_H_2, RORγt and STAT-3 for T_H_17, PU-1 for T_H_9 [8], BCL6 for T_FH_ [9] and Foxp3 and STAT-5 for nT_REG_ and iT_REG_ cells. Although Bcl6 and PU-1 are necessary for T_FH_ [9] and T_H_9 [8] cell differentiation, respectively, they are not sufficient to coordinate the full transcriptional programme, suggesting that other, or additional transcriptional regulators are required. The TF Foxp3 appears to be restricted to T_REG_ cells [10] and is essential for the development, maintenance and function of T_REG_ cells [11–13]. Deficiency in Foxp3 can lead to severe immunopathology with multi-organ lymphoproliferative autoimmune disease identified in spontaneous mutant *scurfy* mice and in rare cases in humans, known as IPEX syndrome (immune dysregulation, polyendocrinopathy, enteropathy, X-linked). For these reasons, Foxp3 has been considered as a master regulator of T_REG_ cell development and function, and is often used as a marker of T_REG_ cells. However, evidence is emerging that Foxp3 alone is not sufficient to regulate the T_REG_ cell phenotype. A combination of computational network inference and proteomics has characterized the highly regulated transcriptional network of co-factors interacting with Foxp3 that are required for T_REG_ cell differentiation [14,15]. Additionally, analysis of genome-wide binding sites and DNAse I sites revealed Foxp3 functions through pre-existing enhancers already bound by co-factors [16], and requires the establishment of a CPG hypomethylation pattern at the Foxp3 binding site [17]. As discussed by others [18], these studies highlight the complexity of signals required for T-cell differentiation, perpetuating the question of adaptation of T_REG_ cells.

**Figure 1.**
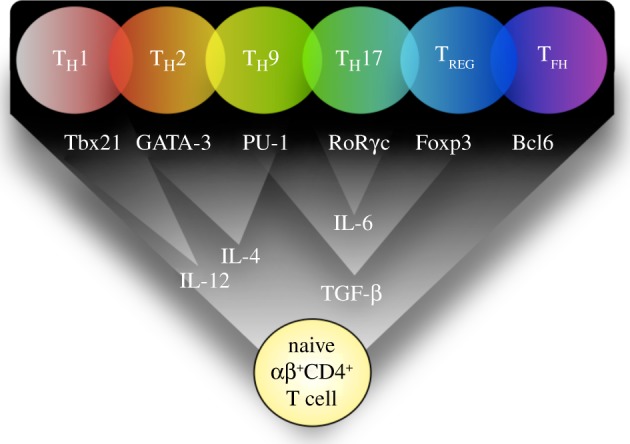
T-cell differentiation pathways. Following TCR ligation with appropriate co-stimulation, cytokines activate specific TFs and transcriptional regulators resulting in the differentiation of T cells into various identifiable states. For example IL-4 activates STAT-6 and GATA-3, initiating and repressing a suite of genes characteristic of T_H_2 cells.

Until recently, the doctrine that αβ^+^CD4^+^ T cells were restricted to a particular fate (including T_H_1, T_H_2, T_H_9, T_H_17, T_FH_ or T_REG_; figure 1) was widely, but not completely, accepted. While the single-fate model is useful, it is often based on *in vitro* studies, often using supra-physiological stimulation, mitogens, phorbol esters and calcium ionophores or high levels of antigen. Recent studies challenging the single-fate model have highlighted a significant degree of flexibility and plasticity between T-cell destinies *in vitro* and to a lesser extent *in vivo*. In this review, we summarize the recent literature reporting T-cell plasticity within and between T_H_, T_FH_ and T_REG_ cells, describe the current proposed mechanisms, and finally ask whether plasticity within αβ^+^CD4^+^ T cells is beneficial or detrimental to immunity.

## 3. The changing profile of helper T cells

### 3.1. T_H_17/T_H_1 conversion

**Figure 2.**
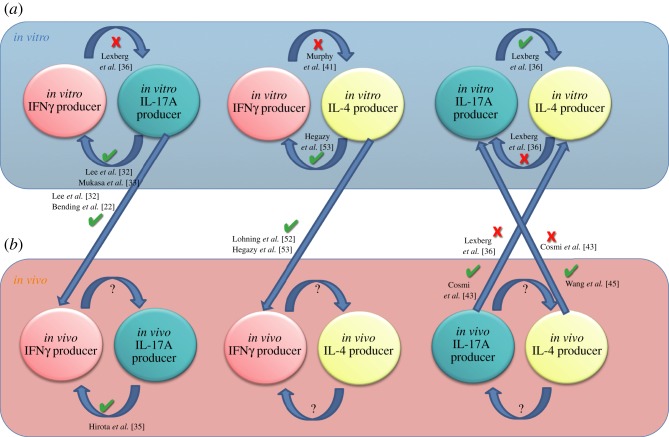
T helper cell plasticity. Several studies have demonstrated the ability of cytokine-producing cells to change their cytokine-producing profile, under various conditions. *In vitro* generated (*a*) IL-17A-producing cells can upregulate IFNγ following re-polarization with IL-12, or following adoptive transfer into mice, as indicated. Similarly, cells that have previously activated an Il-17a programme *in vivo* (*b*) can upregulate IFNγ during EAE, as indicated. Whether other cytokine-producing cells display similar plasticity *in vivo* has not been conclusively demonstrated.

Since the identification of IL-17A-secreting T_H_17 cells almost a decade ago [19] and the later discovery of the signals required for their development [20,21], T_H_17 cells have been found to be relatively unstable [22,23], with IL-4 [24], IFNγ [25,26], high-dose TGF-β [21], IL-2 [27] and IL-27 [28] all capable of inhibiting or suppressing T_H_17 cell differentiation (figure 2). *In vitro* and *ex vivo* from mice [29,30] and humans [31], IFNγ and IL-17A co-producing cells were evident, but largely ignored. Addressing this phenomenon in more detail, Lee *et al*. [32], and later Mukasa *et al*. [33], reported that cells polarized under T_H_17 conditions *in vitro* were capable of producing IFNγ upon secondary culture in T_H_1 conditions, including IL-12 and blocking antibodies against IL-4. This was not simply an *in vitro* phenomenon, as *in vivo* adoptively transferred T_H_17 cells were able to upregulate and produce IFNγ during colitis [32,34] or in nucleotide oligomerization domain/severe combined immunodeficiency (NOD/SCID) mice [22]. Whether T_H_1, T_H_17 or an independent pathway gave rise to IFNγ^+^IL-17A^+^ cells was unclear. Given that IFNγ can suppress T_H_17 cells [25,26], it stood to reason that IFNγ^+^ IL-17A^+^ cells originated from T_H_17 cells. Recently, Hirota *et al*. [35] generated an IL-17A fate reporter mouse allowing the accurate fate-mapping of cells that had transcribed *Il17a* and thus been through a T_H_17 programme. Using these fate-mapping mice in a model of multiple sclerosis, experimental autoimmune encephalomyelitis (EAE), the authors demonstrated that the majority of pathogenic IFNγ-secreting cells had, at some point, derived from T_H_17 cells [35], supporting previous studies [22,32,36,37]. In contrast to the EAE model, Hirota *et al*. [35] further demonstrated that IFNγ-secreting T_H_1 cells developed independently from T_H_17 cells following acute cutaneous infection with *Candida albicans*. It remains unclear whether the difference in conversion reflects a distinction between chronic inflammation (in the EAE model) and acute inflammation (following *C. albicans infection*), as suggested by the authors, or between autoreactivity and immunity to infection. Feng *et al*. [34] also identified the conversion of T_H_17 to T_H_1 cells *in vivo*. Mechanistically, the authors identified that IL-17A induced IL-12 secretion from innate cells, facilitating the conversion of T_H_17 cells to T_H_1 during experimental colitis. To date, it appears that under appropriate conditions T_H_17 cells can upregulate T_H_1 features, including Tbet expression and IFNγ secretion. There is limited evidence to suggest the contrary, that T_H_1 cells can adopt a T_H_17 phenotype whether *in vitro* or *in vivo*. For example, *in vitro* studies found that polarized T_H_1 cells do not readily upregulate RORγt or produce IL-17A when re-cultured in T_H_17-polarizing cocktails [36]. This may be due to downregulation of the IL-6 receptor on activated T cells [38], a critical component of the T_H_17-polarizing cytokine cocktail. *In vivo*, however, this could be overcome through IL-6 presented *in trans*, bound to IL-6R^+^ cells, or in complex with soluble IL-6R [39]. Nevertheless, T_H_1 conversion to a T_H_17 phenotype does not appear to occur in C57BL/6 mice.

### 3.2. T_H_17/T_H_2 conversion

Similar to T_H_1 and T_H_17 cells, there is evidence of cross-regulation between T_H_2 and T_H_17 subsets, with T_H_2-derived IL-4 capable of inhibiting initial T_H_17 differentiation [25] and subsequent IL-17A secretion from committed T_H_17 cells [24] (figure 2).

Interestingly, cells undergoing repeated rounds of stimulation in T_H_17-polarizing conditions *in vitro* become resistant to the suppressive effects of IL-4, indicating that mature T_H_17 cells become more rigid or stable.

*In vitro-* or *ex vivo-*derived T_H_17 cells, sorted by fluorescence activated cell sorting using an IL-17A cytokine secretion assay, could produce IL-4 upon secondary culture in T_H_2 conditions, or upon transfer into helminth-infected mice [40], suggesting that IL-4-sensitive T_H_17 cells can actively convert into IL-4-secreting T_H_2 cells. A separate study suggested that T_H_17 cells were more rigid, with IL-17A-producing T cells isolated *ex vivo* refractory to T_H_2 conversion when re-stimulated with IL-4 [36]. Whether the stage or maturity of T_H_17 differentiation, as suggested above [41], antigen exposure and specificity or receptor expression distinguishes these studies was unclear from the reports. The hypothesis that T_H_17 cells can convert to T_H_2 cells is further supported by *in vivo* observations, mainly in the context of lung inflammation [42,43]. IL-13^+^IL-17A^+^ CD4^+^ T cells were observed in the lungs and draining lymph nodes of mice following repeated administration of ovalbumin (OVA)-pulsed dendritic cells. Co-culture of OVA-pulsed dendritic cells with *in vitro*-polarized T_H_17, but not T_H_2, cells led to the development of an IL-17A^+^IL-13^+^ T_H_ population, indirectly suggesting that at least in this model T_H_17 cells could take on a T_H_2-like phenotype, but that T_H_2 cells could not adopt a T_H_17-like phenotype [42].

*In vitro* observations also support the notion that T_H_17 cells can be re-programmed into T_H_2 cells, but not vice versa [36]. The transcriptional repressor growth factor independent 1 (Gfi-1) can partially explain the lack of T_H_2 to T_H_17 conversion. Gfi-1 is induced by IL-4, stabilizing T_H_2 cells. However, Gfi-1-deficient T_H_2 cells were able to produce IL-17A in secondary T_H_17 culture conditions [44]. The authors elucidated, through chromatin immunoprecipitation (CHIP) analysis, that Gfi-1 modifies T_H_17-associated genes, *Rorc* and *Il23r*, preventing their transcription. Thus, activation and IL-4-induced Gfi-1 in T_H_2 cells serves to promote T_H_2 cell differentiation and prevent T_H_17-associated gene transcription. IL-17A^+^IL-4^+^ double-producing cells have also been observed within the CCR6^+^CD161^+^CD4^+^ population in humans. Notably, IL-17A^+^IL-4^+^ cells were increased among patients with chronic asthma. Culturing human memory T_H_17 cells with IL-4 led to the induction of IL-17A^+^IL-4^+^ cells, while culturing T_H_2 clones with IL-23 and IL-1β did not [43], similar to the murine studies mentioned above. In contrast, one study identified that IL-17A^+^IL-4^+^ memory CRTH2^+^CCR6^+^CD4^+^ cells could be generated from ‘T_H_2’ (CCR6^–^CRTH2^+^CD4^+^) cells in the presence of IL-1β, IL-6 or IL-21 (or most potently, a combination of all three cytokines and *not* IL-23). If CCR6^–^CRTH2^+^CD4^+^ cells are bona fide T_H_2 cells, then this study indicates that T_H_2 cells are capable of adopting a T_H_17 profile [45]. The overwhelming evidence from both human and murine studies indicates that T_H_17 cells, either generated *in vitro* or *in vivo*, can adopt a T_H_2 phenotype whether re-cultured *in vitro* or adoptively transferred *in vivo*, with less evidence to support T_H_2 conversion into T_H_17 cells.

### 3.3. T_H_1/T_H_2 conversion

The relationship between T_H_1 and T_H_2 cells has been the subject of a vast amount of research. Notably, there is much evidence to suggest that T_H_1 and T_H_2 cells cross-regulate one another (figure 2). For example, *in vitro* studies show that T_H_2-associated GATA-3 inhibits T_H_1-related IFNγ [46] and T_H_1-associated Tbet inhibits T_H_2-related GATA-3 [47]. It has also been demonstrated that after repeated rounds of stimulation *in vitro*, T_H_1 and T_H_2 cells lose their ability to interconvert [41]; that is, T_H_1 and T_H_2 cells are less plastic following more rounds of cell division [48]. One simple explanation for this is the downregulation of IL-12Rβ expression on T_H_2 cells that was shown *in vitro* [49], rendering T_H_2 cells un-responsive to lL-12; however, this has been later challenged [50].

Furthermore, *in vitro* cells may be substantially different from *in vivo* cells, as IFNγ^+^IL-4^+^ cells can be readily observed *in vivo* in mice [51]. As a proof-of-principle using murine transgenic TCR-restricted T cells, *in vitro-*polarized, lymphocytic choriomeningitis virus (LCMV)-specific T_H_1 or T_H_2 cells could give rise to comparable frequencies of IFNγ-producing cells following LCMV infection. Interestingly, the T_H_2-polarized cells gave rise to a substantial population of cells co-expressing IL-4 and IFNγ [52]. The conversion of LCMV-specific T_H_2 cells required TCR stimulation as well as the presence of type I and type II interferons [53]. The authors also report a substantial population of IFNγ-producing cells developing from *in vitro*-derived T_H_2 cells when cultured in secondary conditions containing IL-12, IFNγ and IFNα/β [53]. In these studies, it is possible that not all adoptively transferred *in vitro* T_H_2 cells were fully committed T_H_2 cells and that TCR-restricted T cells do not reflect natural polyclonal T-cell populations. Nevertheless, these data not only highlight the ability of T_H_2 cells to become IFNγ-secreting cells, but also highlight that factors present *in vivo*, which are not common constituents of *in vitro* culture systems, such as type 1 interferons, can clearly contribute to T_H_ plasticity.

### 3.4. IL-9-secreting T cells (T_H_9)

In addition to the ability of T_H_2 cells to co-express IFNγ, two reports independently identified the secretion of IL-9 from T_H_2 cells and suggested that T_H_2 cells could be re-programmed to produce IL-9. These reports led to the classification of T_H_9 cells. These initial studies used IL-4^gfp^ reporter mice to generate T_H_2 cells *in vitro* and subsequently identified that TGF-β provided an essential conversion signal to IL-4^gfp+^ cells. ‘Ex- T_H_2’ cells downregulated classical T_H_2 genes (*Gata3 and Il4*) and upregulated IL-9 [54,55]. The T_H_2 heritage of IL-9-secreting cells is supported by their requirement for STAT-6 [56,57] and the observation of IL-9-producing T cells in T_H_2-associated allergic inflammation [58–60]. However, T_H_9 cells have also been identified in autoimmunity [61] and more recently in *Mycobacterium tuberculosis* infection [62], more commonly associated with T_H_1/T_H_17 responses. Whether IL-9-secreting cells are indeed a distinct lineage [63], warranting a ‘T_H_’ prefix, or simply recently activated T_H_, as suggested by others [64], or T_REG_ cells [65] remains to be clarified. Candidates for a T_H_9 ‘master regulator’ have been suggested, however, including PU-1 [8]. Thus, whether IL-9 secretion by T_H_1, T_H_2, T_H_17 or T_REG_ cells constitutes T-cell plasticity or not is unclear at present.

In summary, the ability of T_H_1, T_H_2 or T_H_17 cells to co-express IFNγ, IL-4, IL-17A or IL-9 can be demonstrated *in vitro* and in more restricted and occasionally contrived situations *in vivo*. Interestingly, these phenomena have most frequently been observed during hyper-inflammatory disorders, such as autoimmune or allergic pathologies, with the exception of the LCMV studies [52,53]. There is little evidence that T_H_ plasticity is beneficial during immunity to infection, and it could be hypothesized that the occurrence of plasticity contributes to the development of inflammatory disorders.

## 4. The changing profile and nature of regulatory T cells

The stability of Foxp3^+^ T_REG_ cells has been, and continues to be, enthusiastically debated, especially as T_REG_-based therapies move closer to the clinic [66–68]. Two novel areas of T_REG_ cell biology, T_REG_ specialization and T_REG_ instability, are fuelling the debate on T_REG_ plasticity. In an attempt to reconcile the debate, Miyao *et al*. [69] developed an innovative Foxp3^GFPCre^ROSA26^RFP^ reporter mouse, which allowed the authors to fate-map cells that had previously expressed Foxp3 (RFP^+^) in addition to identifying those cells currently transcribing Foxp3 (GFP^+^). Through a series of adoptive transfer experiments, the authors propose a heterogeneity model identifying populations of both unstable ‘exFoxp3^+^’ cells which transiently upregulate Foxp3 following activation without adopting suppressor function (Foxp3^+^ non-T_REG_ cells) and populations of stable Foxp3^+^ T_REG_ cells. The authors also identify that in the periphery, unstable Foxp3^+^ cells were CD25^–^ or CD25^lo^, whereas more stable Foxp3^+^ T_REG_ cells were CD25^hi^. Nevertheless, there is substantial evidence that Foxp3^+^ T cells, whether CD25^hi^ or CD25^int^, that have lost Foxp3 expression adopt important biological functions, which we summarize below [70]. It is important to note that some of the studies described may be compromised by the use of the Foxp3^gfp^(Foxp3^tm2Ayr^) reporter knockin mice. In two separate observations, the EGFP–Foxp3 fusion was shown to disrupt the transcriptional landscape of the T_REG_ cell and therefore affect both the frequency of T_REG_s and their suppressive properties [71,72]. We indicate, where possible, in the studies mentioned below whether inducible or natural T_REG_ cells were studied; however, in many cases it was not always clear.

### 4.1. T_REG_ specialization: co-expression of multiple transcription factors

Recent studies have revealed that multiple TFs are co-expressed in Foxp3^+^ T_REG_ cells, and essential for T_REG_ function, including several TFs associated with T_H_ cell phenotypes. For example, Koch *et al*. [73] identified a population of Foxp3^+^ T_REG_ cells that co-expressed the T_H_1-associated TF Tbet and the chemokine receptor CXCR3 during *M. tuberculosis* infection in mice. Functionally, Tbet expression in T_REG_ cells was required for the proliferation of T_REG_ cells *in vitro* and *in vivo*. Concordant with this, Tbet-deficient T_REG_ cells transferred into scurfy mice were unable to control T_H_1 cells. This phenomenon of IFNγ-secreting Foxp3^+^ cells is further supported and extended in a recent study identifying that IFNγ secretion by Foxp3^+^ cells was necessary for their regulatory function in a model of graft-versus-host disease [74,75].

**Figure 3.**
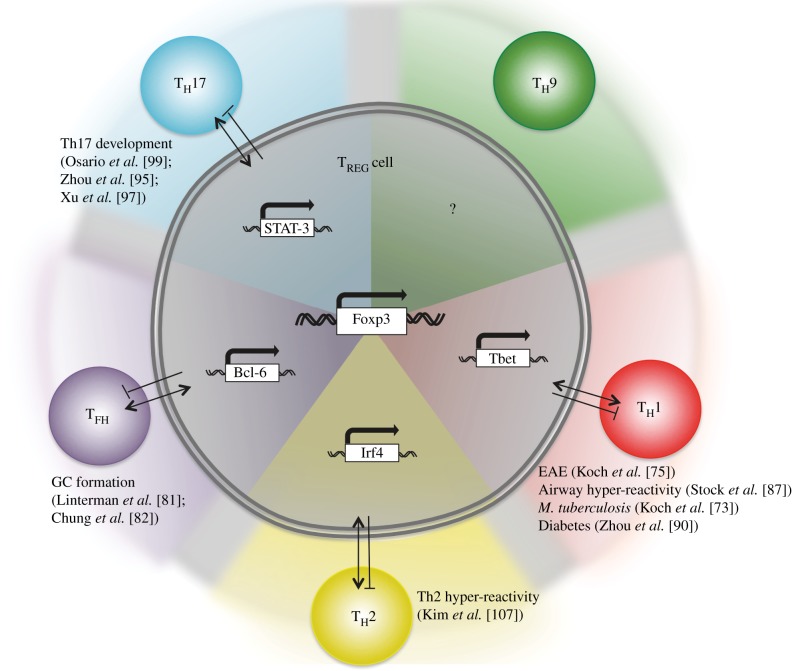
T_REG_ specialization and plasticity. T_REG_ cells can co-express T helper cell lineage-defining TFs, such as Tbet and Foxp3 (red, lower right segment), during various infectious or inflammatory scenarios. This specialization appears to fine tune T_REG_ cells to more effectively regulate the corresponding effector T_H_ cell. For example Tbet^+^Foxp3^+^ T_REG_ cells can potently suppress Tbet^+^ T_H_1 cells. Thus, the co-expression of various TFs is required to confer the appropriate and necessary regulatory programme. Whether these hybrid ‘specialized’ T_REG_ cells are intermediate cells in between the T_H_ to T_REG_ conversion (indicated by arrows in figure), or a stable population is unclear. GC, germinal centre.

Similarly, IRF4, a TF involved in several T_H_ cell subsets, particularly T_H_2 and T_H_9 cells [21,76], has been identified in Foxp3^+^ T_REG_ cells. Significantly, mice lacking *Irf4* in Foxp3^+^ T_REG_ cells failed to control spontaneous T_H_2-mediated pathologies [77]. Further work from the Rudensky laboratory identified that STAT-3, a TF required for T_H_17 cells [78], was required for Foxp3^+^ T_REG_ cells to control T_H_17 cells in mice [79], confirming previous *in vivo* observations identifying the requirement of STAT-3 for T_REG_ function [80]. Finally, T_FH_ cells are also regulated by a subset of specialized Foxp3^+^ T_REG_ cells that co-expressed Bcl6, the same TF required for T_FH_ cell development [81,82] (figure 3). Interestingly, Cipolletta *et al*. [83] describe a specialized population of Foxp3^+^ T_REG_ cells resident in visceral adipose tissue (VAT) expressing the nuclear receptor peroxisome proliferator-activated receptor (PPAR)γ. These T_REG_ cells play a unique role in suppressing obesity-induced VAT inflammation; however, the mechanism of suppression by these T_REG_ cells is still unclear. Collectively, these studies indicate that T_REG_ cells become functionally specialized to control distinct T_H_ and T_FH_ responses, and perhaps in response to cues from distinct anatomical sites. Secondly, these studies show that T_REG_ cells co-opt similar TF-dependent pathways to the T_H_ cells they regulate. Of note, GATA-3 expression has also been widely reported in Foxp3-expressing cells [84]; however, unlike the focused T_H_1-, T_H_2-, T_H_17- or T_FH_-controlling Foxp3^+^ cells described above, GATA-3 was broadly required for stable Foxp3 expression and general T_REG_ function [85,86].

## 5. T_REG_ instability: conversion to T effector phenotypes

### 5.1. T_REG_/T_H_1 conversion

The relationship between T_H_1 and T_REG_ was first described in a study that identified a population of OVA-specific T_H_1-related Foxp3^+^ T_REG_, which produced IL-10 and IFNγ, co-expressed Tbet and Foxp3 and had the capacity to suppress allergen-induced airway hyper-reactivity [87]. The ontogeny of Tbet^+^Foxp3^+^ cells in this study, as in others, was unclear. Evidence of Foxp3^+^ T_REG_ cells converting into IFNγ-producing T_H_1 cells has been reported in several systems. Firstly, Foxp3 deletion in mature T_REG_ cells *in vivo* led to the development of pro-inflammatory T_H_ cells secreting IL-2 and IFNγ [88], indicating that Foxp3^+^ actively represses Tbet and a T_H_1 programme. Functionally, transfer of these Foxp3-deficient ‘T_REG_’ cells into lymphopenic hosts led to severe autoimmunity, indicating that these cells acquired pathogenic potential and retained self-antigen specificity [88]. In a separate study, 50 per cent of adoptively transferred natural Foxp3^+^ T_REG_ cells transferred into lymphopenic mice lost Foxp3 expression and up to 25 per cent started producing tumour necrosis factor (TNF)-α, IFNγ or IL-4 [89]. Similarly, Zhou *et al*. [90] identified a population of unstable Foxp3^+^ cells in healthy mice that adopted a T_H_1-like phenotype and were partially responsible for islet cell destruction and the development of diabetes. Collectively, these studies indicate that during lymphopenia [89], Foxp3 deletion [88] or autoimmunity [90], a fraction of T_REG_ cells could acquire a pro-inflammatory IFNγ-secreting phenotype. Similarly, during lethal enteric *Toxoplasma gondii* infection, Foxp3^+^ cells lost their T_REG_ phenotype and converted into pathogenic IFNγ-secreting cells [91]. The conversion of T_REG_ cells into IFNγ^+^ cells, but not IFNγ^+^ cells into Foxp3^+^ cells, is supported by a study by Feng *et al*. [92] who identified that microbiota antigen-specific inducible Foxp3^+^ T_REG_ cells could upregulate IFNγ in response to the T_H_1-polarizing cytokine IL-12 [92]. Furthermore, these IFNγ^+^Foxp3^+^ cells retained regulatory properties, before full conversion into pathogenic, non-regulatory, IFNγ^+^ cells. In both of these studies, IL-12 was identified as a critical component of IFNγ production by Foxp3^+^ cells.

In humans, although Foxp3 is not an exclusive marker of T_REG_ cells [93], a population of human CD4^+^CD127^lo^CD25^+^ T cells, which expressed Foxp3, were found to produce IFNγ. These putative regulatory cells were present at higher levels in patients with type 1 diabetes and possessed mild suppressive properties, although reduced suppressor function compared with IFNγ^–^T_REG_ cells [94]. Whether Foxp3 expression was only transiently expressed, a feature common to recently activated human T_H_ cells [93], or stably expressed in a T_REG_ cell was unclear in this study. Collectively, these murine and human studies suggest that T_REG_ cells, which maintain peripheral tolerance, can convert into pathogenic T_H_1-associated cells capable of causing autoimmunity and lethal inflammation. The mechanisms for conversion have not been completely elucidated in these systems. It is not yet clear whether plasticity in various systems relies on common mechanisms or is specific to the local micro-environment. Potential mechanisms of plasticity are discussed later in this review.

### 5.2. T_REG_/T_H_17 conversion

The reciprocal relationship between IL-17A-secreting RORγt^+^ cells and inducible Foxp3^+^ T_REG_ cells has been widely reported. For example, TGF-β promotes the expression of both Foxp3 and RORγt . However, Foxp3 directly inhibits RORγt *in vitro* leading to a regulatory T-cell phenotype [95]. The initial observation that innate cell-derived IL-6 could block TGF-β-mediated iT_REG_ induction and iT_REG_-mediated suppression [76] raised the possibility that iT_REG_ cell development or function could be interrupted by inflammatory cytokines. Several years later, two independent groups [20,21] identified that IL-6 and TGF-β induced T_H_17 differentiation, providing a divergent molecular mechanism of iT_REG_ and T_H_17 development. Thus, TGF-β in the presence or absence of IL-6 [96] can act as a critical tipping point directing the development of T_H_17 or T_REG_ cells, respectively. The balance between iT_REG_ and T_H_17 cells may be intricately regulated as Foxp3^+^ T_REG_ cell-derived TGF-β [97] and T_REG_-induced IL-6 from mast cells [58] can promote de novo T_H_17 differentiation in naive T cells.

Several reports have identified cells *in vivo* co-expressing RORγt and Foxp3 [95,98] with the ability to differentiate into pathogenic RORγt^+^Foxp3^+^IL-17A^+^ [99] or regulatory RORγt^+^Foxp3^+^IL-10^+^ [98] cells. The developmental crossroads may be regulated by IL-6 or other innate cytokines as rIL-6-exposed Foxp3^+^ T_REG_ cells can upregulate IL-17A *in vitro* [97]. Whether *in vivo* Foxp3^+^ T_REG_ cells are similarly responsive to IL-6, and IL-12 as described above [57] remains to be demonstrated. The clearest description of IL-17A-producing T cells developing from a Foxp3^+^ source was identified using fate-mapping Foxp3^Cre^ mice, labelling cells that had previously transcribed *Foxp3*. In this study, 22 per cent of IL-17A-producing cells in the small intestine had expressed Foxp3 at some point in their development [90].

In addition to IL-6, which can function as a molecular switch between iT_REG_ and T_H_17 cell differentiation, as described above, Sharma *et al*. [100] identified that indoleamine 2,3-dioxygenase (IDO) [101], a tryptophan-catabolizing enzyme produced by plasmacytoid dendritic cells (pDCs) and potentially other cells, maintains the T_REG_/T_H_17 balance in tumour-draining lymph nodes by regulating IL-6 production. Inhibition of IDO led to increased IL-6 and the conversion of Foxp3^+^ T_REG_ cells into polyfunctional IL-2, TNF-α, IL-22 and IL-17A-secreting cells. Similar T_REG_ to T_H_17 conversions have been observed in human T cells, with T_REG_ cells cultured *in vitro* with IL-2 and IL-15 losing Foxp3 expression and secreting IL-17A, IL-22, IFNγ and IL-21 [59]. Using T_REG_ cell clones, Beriou *et al*. [102] were able to further demonstrate that Foxp3^+^ IL-17A^+^ T_REG_ cells retained the capacity to suppress or secrete IL-17A, depending upon the stimulation. Foxp3^+^ IL-17A^+^ clones stimulated with IL-1β and IL-6 produced IL-17A, whereas Foxp3^+^IL-17A^+^ clones treated with IL-2 were potent suppressive cells [102], suggesting a dynamic switch between regulatory and effector functions in response to environmental cytokines.

Foxp3^+^CD25^+^CD45RA^+^CCR6^+^ cells that co-express RORγt, with the capacity to secrete IL-17A following re-stimulation with phorbol 12-myristate 13-acetate/ionomycin or pro-inflammatory cytokines IL-1β, IL-6, IL-2, IL-21 and IL-23 have also been identified in the peripheral blood [103] and tonsils [104] of healthy donors. These cells were also able to suppress CD4^+^ T cells via cell contact-dependent mechanisms. Given the close developmental relationship between iT_REG_ and T_H_17 cells [95] and the intimate cross-regulation by RORγt and Foxp3, the conversion between T_H_17 and T_REG_ cells may not be too surprising. However, the opposing function of these cell types would require tightly regulated mechanisms, critical to preventing regulators of autoimmunity converting into effectors. Whether a breakdown in these regulatory pathways, such as the IDO/IL-6 pathway described above [100], underpins the development of autoreactivity, in addition to tumour immunosurveillance, is unclear.

### 5.3. T_REG_/T_H_2 conversion

The ability of T_REG_ cells to convert into IL-4-secreting T_H_2 cells has also been reported. The Foxp3^IRES-luciferase-IRES-eGFP^ (FILIG) mouse, which has a 5–10% reduction in Foxp3 expression in CD4^+^ T cells, develops an aggressive autoimmune disorder and wasting disease. Interestingly, cells from FILIG mice that had reduced Foxp3 expression lost their suppressive activity and started producing T_H_2 cytokines, including IL-4 and IL-13, and to a lesser extent IL-2, IFNγ and IL-17A [105], similar to Foxp3-ablated T_REG_ cells [88]. More conclusively, adoptive transfer of FILIG T_REG_ cells, with attenuated levels of Foxp3, into TCRα^–/–^ or RAG2^–/–^ mice preferentially differentiated into T_H_2 cells and produced IL-4 [106]. Mechanistically, T_REG_ to T_H_2 cell conversion was dependent on GATA-3 and independent of STAT-6 signalling. However, for stable IL-4 production by ‘exFoxp3’ cells an IL-4/STAT-6/GATA-3 loop was required [85,106]. There may be a dynamic relationship between T_H_2 and T_REG_ cells, as T_H_2 cells stimulated with TGF-β, retinoic acid and antibodies to IL-4 and IFNγ *in vitro* downregulated T_H_2 signature genes, lost production of IL-4 and IL-13 and adopted a Foxp3^+^ regulatory phenotype [107]. Furthermore, these converted T_H_2-derived memory Foxp3^+^ T cells could suppress T_H_2-mediated airway hyper-reactivity when adoptively transferred *in vivo*, suggesting that the converted ex-T_H_2 cells could gain not only Foxp3 expression but also suppressive function [107].

### 5.4. T_REG_/T_FH_ conversion

Finally, the plasticity or transient nature of Foxp3 expression in some T_REG_ cells permitted the conversion of T_REG_ cells to T_FH_ cells. Under lymphopenic conditions, adoptively transferred Foxp3^+^ T_REG_ cells downregulated Foxp3 expression in the Peyer's patches clustered around germinal centres and expressed T_FH_ cell-associated markers CXCR5, IL-21 and Bcl6 [108]. As described above, specialized T_REG_ cells that upregulated Bcl6 and CXCR5 acquired the ability to preferentially regulate T_FH_ cells [81,82]. Whether some T_FH_ cells retain plasticity, with the ability to self-regulate by upregulating Foxp3, or whether all three populations (T_FH_, T_REG_ and T_FH_/T_REG_) develop independently is unclear.

In summary, it is clear that some, possibly CD25^–^ or CD25^lo^ Foxp3^+^, cells [69] display elements of plasticity; losing Foxp3 expression and adopting helper or follicular helper phenotypes with distinct cytokine-producing capacity. In the light of the recent study by Miyao *et al*. [69], whether exFoxp3 cells described above originate from peripheral Foxp3^+^CD25^–^ or Foxp3^+^CD25^lo^ populations, with variable IL-2-responsiveness, or not is unclear. These data would imply that IL-2 signalling in T_REG_ cells is not only required to maintain T_REG_ stability, but also to prevent plasticity and T_H_ cell conversion. In keeping with this, *in vivo* IL-2 blockade resulted in a loss of peripheral Foxp3^+^ cells and the development of autoimmune gastritis [109]. Whether the pathogenic T cells, which caused gastritis in this model, originated from a Foxp3^+^ population upon IL-2 depletion was unclear.

## 6. Potential mechanisms of T-cell plasticity

**Figure 4.**
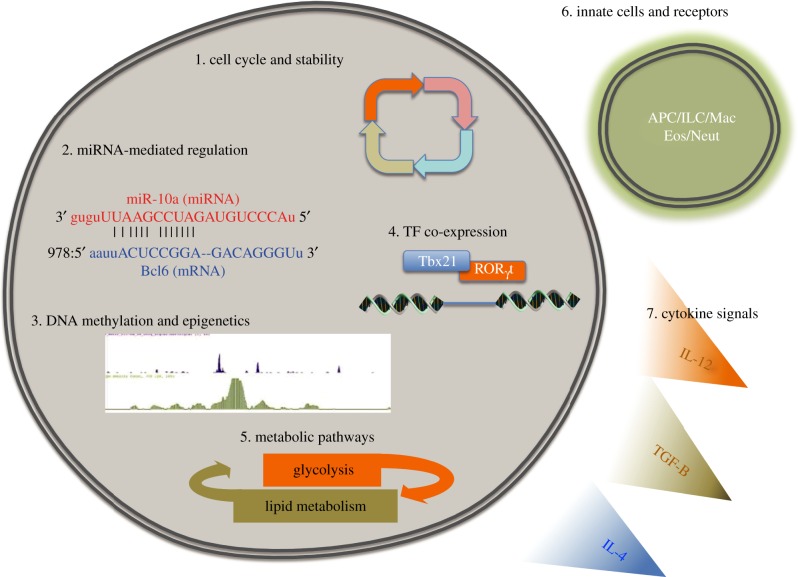
Potential mechanisms of T-cell plasticity. Various mechanisms of T-cell plasticity have been tested, suggested and loosely implied. Intrinsic mechanisms, (1) including the stage of T_H_ cell maturation may be inversely correlated to plasticity. (2) Post-transcriptional regulation by small RNA molecules, including miRNAs, can dramatically alter the T-cell phenotype. (3 and 4) Changing TF expression and activation with permissive epigenetic marks at TF binding sites can re-programme entire gene programmes. (5) A change in nutrient availability may trigger changes in intracellular metabolic pathways and the resultant T-cell phenotype and function. (6 and 7) Extracellular influences, including interactions with innate cell receptors or triggering of cytokine signalling pathways may dynamically alter cytokine receptor expression on T cells, making them permissive to subsequent re-programming signals. APC, antigen-presenting cell; Eos, eosinophil; ILC, innate-like helper cells; Mac, macrophage; Neut, neutrophil.

From the studies mentioned above, the ability of CD4^+^ T cells to change their phenotype is clear. Whether there is progression from a less stable to a more stable state, as suggested by others [110], or whether the T-cell phenotype is simply a reflection of the transient micro-environment has yet to be determined. Although not directly tested in any of the studies mentioned throughout this review, whether the genetic background of mice used contributes to plasticity or not is unclear and yet to be tested. With the advent of well-defined genetic tools, such as the international Collaborative Cross [111], dissecting genetic determinants of T-cell responsiveness will now be much easier. However, to date, several mechanisms that influence T-cell plasticity have been proposed, generally separable into extrinsic and intrinsic pathways (see figure 4).

## 7. Cell extrinsic mechanisms of T-cell conversion

### 7.1. Accessory innate cells and innate receptors

Although often bypassed using *in vitro* T-cell assays, antigen-presenting cells (APCs) displaying various co-stimulatory molecules on their surface translate innate antigen recognition signals into the appropriate instructions for T cells. It has been well documented that high antigen doses, and higher affinity peptides, polarize responding naive T cells into T_H_1 cells, while low antigen doses, and lower affinity peptides, favour T_H_2 polarization [112,113]. It is therefore conceivable that the T_H_ cell response may transition from a pro-inflammatory T_H_1-, and possibly T_H_17-, dominant phenotype during antigen abundance, or high pathogen load in the case of infection, when cells are also potentially refractory to T_REG_-mediated suppression [114], into a T_H_2 phenotype as the antigen is reduced. Beyond TCR–major histocompatibility complex II–peptide interactions, co-stimulatory molecules on APCs, particularly the B7 family members, which greatly influence T-cell differentiation [112,115,116], may also have the potential to transform and re-polarize differentiated T_H_ cells by modulating cytokine responsiveness [117]. Through germline encoded receptors, including toll-like (TLR) and NOD-like receptors, APCs can influence the resultant T-cell response. Ligation of specific TLRs on various innate cells elicits divergent co-stimulatory molecule expression and cytokine secretion. This feature of highly responsive innate receptors on APCs is currently being therapeutically targeted to deviate adaptive immune responses during cancer, and infectious and allergic diseases (reviewed by Kanzler *et al.* [118]). For example, treatment of allergen-sensitive mice, which have T_H_2-polarized T_H_ cells, with CpG-oligodeoxynucleotides that stimulate TLR9, downregulated B7.2 (CD86) in lung tissue and deviated T_H_2 responses towards T_H_1 responses [119]. Whether TLR9 ligation on APCs relayed a signal to convert T_H_2 cells into T_H_1 cells was not explored. Furthermore, T cells themselves possess the same germline-encoded innate recognition receptors as innate cells. *In vitro* TLR4 ligation on T_H_ cells during T-cell differentiation did not preferentially alter T_H_1, T_H_2, T_H_17 or iT_REG_ cytokine responses, but prolonged survival and expansion, suggesting a common TLR4-driven signalling pathway in T_H_ cell subsets [120]. However, *in vivo* experiments highlighted the requirement of TLR4 ligation for T_H_1 and T_H_17-mediated disease. Disruption of TLR signalling, by deleting the essential downstream adaptor MyD88 in T cells, compromised protective T_H_1-mediated immunity to *T. gondii* [121]. Using an EAE model and TLR4 [120] or TLR2-deficient [122] CD4 T cells, T_H_17 and T_H_1-dependent disease was also significantly abrogated. Further support for TLR4 signalling in T cells has been reported in a model of colitis [123], where TLR4/IL-10-deficient T cells were more pathogenic, compared with IL-10-deficent cells. Although the extent of TLR signalling on T-cell stability and plasticity has not been reported, given the requirement for TLR4-mediated signals for T_H_17 and T_H_1 responses, TLR signalling could be an influential trigger in T-cell phenotype decisions.

Other innate cells, including IL-4-secreting basophils, neutrophils in various stages of apoptosis and inducible nitric oxide-producing macrophages, can promote T_H_2 [124,125], T_H_17 [126] or T_H_1 [127] differentiation, respectively, and may also contribute to T-cell plasticity. Finally, the emerging field of innate-like helper cells (ILCs), which appear to mirror T_H_ cell subsets [128], can influence naive T-cell differentiation [129], and potentially differentiate T cells promoting plasticity. The high levels of IFNγ, IL-17A and IL-22 or IL-5 and IL-13 secreted by the three main populations of ILCs have the potential to deviate T-cell and non-T-cell responses.

### 7.2. Cytokine micro-environment and cytokine receptor regulation

The cytokine micro-environment can activate, inhibit and directly modify differentiated T_H_ cells. With respect to T-cell plasticity, type-1 IFNs can induce the expression of IL-12R on T_H_2 cells, allowing the necessary IL-12 signals to induce Tbet and IFNγ secretion [53] and subsequent T_H_2 to T_H_1 conversion. This mechanism of type-1 IFN-mediated T_H_2 to T_H_1 conversion via cytokine receptor regulation supports observations made over 10 years ago identifying that IFNγ and IFNα mediate the decay of IL-4R [130]. Regulation of IL-12R and sensitivity to the potent effects of IL-12 [131] and IL-18 [132] has long been appreciated in the differentiation of T_H_1 and T_H_2 cells [49]. Initial studies demonstrated that T_H_2 cells downregulate IL-12R, leaving cells refractory to IL-12, while T_H_1 cells operate positive re-enforcement with IFNγ-mediated STAT-1 activating Tbet and up-regulating IL-12R expression [133]. In our unpublished observations, and reported by others [50], downregulation of IL-12R did not completely abrogate IL-12 signalling in T_H_2 cells. IL-2, an important T-cell growth factor for all other T cells, downregulates IL-7R [134] and IL-6R, and upregulates IL-4R and IL-12Rβ2, inhibiting T_H_17 generation [27] but facilitating T_H_1 and T_H_2 differentiation [135]. Furthermore, IL-2 is tightly regulated in T_H_17 cells by Aiolos, a member of the Ikaros family of TFs [136], preventing IL-2 production and the potential for IL-2 to antagonize T_H_17 development. Similarly, many studies have identified the ability of IL-27 to antagonize T_H_17 differentiation and effector function in a STAT-1-dependent manner [28,137–141] and increase responsiveness to IL-12 [142]. The combined ability of IL-12 signalling to re-direct TGF-β-orchestrated T_REG_ or T_H_17 programmes [131], coupled with multiple pathways regulating IL-12 receptor expression and responsiveness, may explain why T_H_1 cells may be more stable. Thus, the conversion of T_H_17 cells into T_H_1, T_H_2 or T_REG_ cells may involve an IL-2–STAT-5 signal, facilitated by IL-27–STAT-1 signals for conversion into T_H_1 cells. Whether canonical cytokine signalling pathways are required for T_H_ cell conversion, such as IL-4, IL-12 and IL-6 for T_H_2, T_H_1 and T_H_17 responses, respectively, is unclear. In the absence of IL-4 and IL-13, T_H_1 cells converted into T_H_2 cells during hookworm infection [40], suggesting that a non-canonical pathway may exist at least for T_H_1 to T_H_2 conversion. Collectively, these studies indicate that the local cytokine environment can modify the expression and responsiveness of various cytokine receptors, rendering differentiated T cells susceptible to alternative differentiation pathways.

### 7.3. Nutrient availability and metabolic pathways

Throughout T-cell development, differentiation and function, metabolic needs are intimately linked [1]. Following activation, helper T cells rapidly upregulate glucose uptake and glycolysis [143,144]. In contrast, regulatory T cells upregulate lipid oxidative metabolism [145], with less glucose uptake and glycolysis. Inhibition of either of these pathways prevents activation, proliferation, cytokine secretion and cellular function [146]. Furthermore, the metabolic needs and pathways of different T_H_ cells diverge, providing another environmental cue that may influence T_H_ cell phenotype switching. For example, distinct phosphoinositide 3-kinase/mammalian target of rapamycin (mTOR) pathways [147], via two mTOR complexes, mTORC1 or mTORC2, are employed by T_H_1 and T_H_17 or T_H_2 cells, respectively [148]. Additionally, small concentrations of the small molecule halofuginone, which induces an amino acid starvation response, can limit T_H_17 but not T_H_1, T_H_2 or iT_REG_ polarization *in vitro* [149]*.* Hypoxia-induced factor (HIF)1α and cMyc, two TFs that regulate glycolysis [150], can also modulate the balance between T_H_17 and T_REG_ differentiation by controlling glycolytic metabolism [151]. Concordantly, mice with HIF1α-deficient T cells, with subsequently compromised glycolysis, have increased T_REG_ cells and are protected from T-cell-mediated autoimmunity [152]. Thus, at the simplest level, shuttling between glycolysis and lipid oxidation pathways can favour T-cell differentiation pathways between T_H_ and T_REG_ cells. It is clear that the T cells have specific metabolic requirements and that these requirements differ between T_H_ and T_REG_ subsets; it is yet undetermined whether these metabolic pathways are important for T-cell plasticity *in vivo*.

## 8. Potential cell-intrinsic mechanisms of T-cell conversion

### 8.1. Cell cycle and phenotype stability

Soon after the description of the T_H_1 and T_H_2 lineages, it was reported that T cells gradually become more fixed in their phenotype after several rounds of differentiation and lose their ability to acquire other T_H_ phenotypes [41,48]. This observation holds true with recent reports identifying that mature T_H_17 cells, compared with immature T_H_17 cells, became less responsive to IL-4 [24]. Together, these studies imply that cytokine positive, early differentiating cells are more plastic than their mature counterparts. Indeed, memory T_H_17 cells were shown to have a stable phenotype [36]. Nevertheless, it has been reported that some antigen-specific memory CD4 cells show substantial plasticity between T_H_1 and T_H_2 phenotypes [153]. Thus, T_H_ plasticity may be intimately linked to not only cell cycle, but also memory status.

### 8.2. microRNA-mediated control of T-cell phenotype

microRNAs (miRNAs) are a family of small non-coding RNAs that provide post-transcriptional regulation of gene expression. There is accumulating evidence that miRNAs are critical in regulating the expression of key molecules in T_H_ and T_REG_ subsets. CD4 T cells deficient in *dicer*, an enzyme required for miRNA biogenesis, had dysregulated cytokine production following *in vitro* culture, including the co-expression of IFNγ and IL-4 in T_H_2 culture conditions [154]. Deletion of another component of the miRNA machinery, *drosha*, specifically in Foxp3-expressing cells resulted in autoimmunity and overexpression of IFNγ and IL-4 [155]. Specific miRNAs that regulate CD4 T-cell phenotypes have also been identified. For example, miR-29, which targets *Tbet*, *Eomesodermin* and *Ifn*γ** [156,157], critically controls T_H_1 cell development. miR-10a regulates Bcl-6 in T_REG_ cells, preventing the development of a T_FH_ cell phenotype from T_REG_ cells [158]. Finally, miR-326 promotes T_H_17 differentiation, with miR-326 expression correlating with disease severity in multiple sclerosis patients [159]. Thus, it is clear that miRNAs are key regulators of T-cell differentiation, and it is likely that miRNAs could regulate both upstream pathways (cytokine receptor, signalling pathways and TF expression) and downstream (effector cytokine production) features of T cells contributing to lineage stability and plasticity, as indicated with miR-10a in T_REG_ cells [158].

### 8.3. Transcription factor dosing and dominance

For TFs to maintain activated and repressed gene programmes, the continuous activation, phosphorylation and presence of TFs in the nucleus is often required. For example in the case of T_REG_ cells, ablation of Foxp3 in T_REG_ cells results in the loss of Foxp3-driven suppressor function [88]. Furthermore, decreased Foxp3 expression converts T_REG_ cells into pathogenic effector cells [105], suggesting that a significant function of Foxp3 is to repress the development of T_H_ cell-associated responses. TFs can also function to reinforce T_H_ phenotypes, as in T_H_1 cells where IFNγ promotes Tbet via STAT-1, which in turn promotes the expression of the IL-12 receptor [132,133]. The importance of TF activation in T-cell phenotypes is supported by forced/ectopic expression experiments. Ectopic expression of Foxp3 in CD4^+^ non-T_REG_ cells leads to acquisition of suppressive function [10,12,160]. Similarly, forced expression of STAT-6 [161], Tbet [162] or RORγt [29] results in T_H_2, T_H_1 or T_H_17 cell development, respectively. Ectopic expression of Tbet in T_H_2 cells results in IFNγ production [101,133], suggesting that Tbet can override the transcriptional programme in T_H_2 cells. Furthermore, there is considerable cross-regulation between TFs in T-cell subsets. For example, Foxp3 can inhibit RORγt function [95], Tbet negatively regulates GATA-3 [47] and GATA-3 downregulates STAT-4 [163]. STAT-5 can also repress the T_FH_ phenotype by suppressing the expression of Bcl-6, among others [164,165]. Thus, a hierarchy of TF expression and activation may ultimately dictate the resultant T-cell phenotype. From these ectopic expression experiments, if sufficient signals induce and activate TFs, then the phenotype of the cell can be re-programmed. It is conceivable, therefore, that modifications of TF expression could be intimately linked with T-cell plasticity. Indeed, it has been shown that in polarized T_H_1 cells, Tbet forms a complex with Bcl-6, preventing its function. Upon limiting IL-2 conditions, the amount of Bcl-6 in the T_H_1 cells increases and the cells are able to express T_FH_-associated genes [166]. Similarly, as described above, expression of Gfi-1 in T_H_2 cells prevents the development of a T_H_17 phenotype; deletion of Gfi-1 allowed T_H_2 cells to adopt a T_H_17 phenotype [44].

The existence of cells co-expressing Foxp3 along with T_H_ cell-associated TFs, including Tbet, GATA-3 or RORγt (described in previous sections), calls into question whether there is a regulated balance between TFs (TF dosage) resulting in either effector, effector/regulatory or regulatory function. Furthermore, the ontogeny of these cells remains to be conclusively clarified, whether dual TF-expressing cells derive from T_H_ or T_REG_ progeny, or independently. If dual TF-expressing T_REG_ cells derive from T_H_ cells, the upregulation of Foxp3 may represent a late stage in T_H_ cell differentiation. In this scenario, ‘ex-T_H_’ cells would retain characteristics of their T_H_ cell past, including antigen-specificity and appropriate homing receptors. The alternative, that dual TF expressing cells originate from a Foxp3^+^ T_REG_ past, is also plausible and has been reported in several experimental systems.

### 8.4. Epigenetic modifications

Recent studies have combined gene expression profiling with ChIP-Seq and high-throughput sequencing to investigate the chromatin state in resting and effector T cells [167,168]. These studies have revealed important insights into the mechanisms of T-cell plasticity and stability. For example, the proximal promoter of *Ifn*γ** has permissive methylation marks in T_H_1 cells, but repressive marks in T_H_2 and T_H_17 cells, indicating that specific effector functions may be regulated through epigenetics. Interestingly, in various T_H_ cells, bivalent marks allowing enhancement or repression were found at TF genes, including bivalent marks at *Tbet* and *Gata3* in T_H_17 cells, at *Gata3* in T_H_1 cells, at *Tbet* in T_H_2 cells, and at *Tbet*, *Gata3*, and *Rorc* in T_REG_ cells. This suggests the potential for substantial reversibility at the TF level [32,168]. T_H_ subsets also show positive marks on the *Bcl-6* locus, providing the possibility for T_H_ cells to take on a T_FH_ phenotype [169]. In addition, studies using both wild-type and STAT-4 or STAT-6 knockout T cells have revealed that these transcriptional regulators have effects on epigenetic modifications in T cells [170]. Given the bivalent marks at TF genes in T_H_ cells, epigenetic modifications of effector genes, such as *Ifn*γ*, Il17a* or *Il5* in T cells may be critical regulators of T-cell effector cytokine production. Although epigenetic modifications influence T_H_ cell gene expression, how epigenetic modifications are regulated in T cells is unclear, and therefore how this mechanism would directly contribute to T-cell plasticity is uncertain.

Multiple overlapping mechanisms may all contribute to T-cell plasticity, including epigenetic modifications, post-transcriptional regulation by miRNAs, changes in metabolic activity and activation of TFs.

## 9. T-cell plasticity in immunity: beneficial or detrimental?

As suggested by others [171], the rapid conversion between T_REG_ and T_H_ cell and within T_H_ cell populations could be a very useful feature of the adaptive immune system. Such dexterity could retain antigen-specificity and subsequent memory, preserve the appropriate tropism and rapidly respond to the changing demands and needs of the local environment. With respect to immunity to infection, we have previously reported that increased resistance to the helminth parasite *Schistosoma mansoni* following drug treatment and IL-10R blockade led to elevated antigen-specific IFNγ, IL-5 and IL-17A production [172]. Similarly, lethal infection of IL-10-deficient mice with the intestinal whipworm parasite *Trichuris muris* led to increased parasite-antigen-induced IFNγ and IL-17A [173]. Whether elevated T-cell-derived IFNγ and IL-17A secretions were from T_H_2 cells (i.e. polyfunctional) or from converted T_H_2 cells (i.e. plasticity) is yet to be determined. Also, the precise involvement of IL-10 in regulating these responses was not investigated. In highly regulated environments such as the gut and airways, an effector response must be able to mature in response to infection and overcome local regulatory mechanisms. Indeed, the ability to mount a rapid and lethal T_H_1 response following oral *T. gondii* infection was due to T-cell plasticity, where Foxp3^+^ cells converted into pathogenic IFNγ-secreting cells [91]. If plasticity contributed to the observed phenotypes following *S. mansoni*, *T. muris* and *T. gondii* infection, then despite providing superior pathogen control, significant immunopathology developed. However, the plasticity of T_H_ cells without severe consequences has also been observed in several infection models [40,51,53,153], indicating that plasticity, when absolutely necessary, can provide T-cell-mediated immunity. It remains unclear when plasticity is required to combat infection, under physiological conditions. Studies in infectious disease models, however, provide ideal systems to probe T-cell plasticity throughout induction, expansion and resolution of the T-cell response. Several studies have identified the plasticity of T cells during autoimmunity [22,35,94] and allergy [42,45,107]. Whether T-cell plasticity contributes to the pathogenesis or resolution of these immunopathologies is too early to tell. Nevertheless, strategies to deviate T-cell responses in allergy are being pursued, as described above [118].

Currently, there is limited evidence showing T_H_ plasticity occurring *in vivo* as part of an effective immune response. Over the coming years, as we move beyond phenomenology, there is a need to ask what proficient T cells do, in addition to what T cells can do when forced *in vitro*. Similarly, the use of a single primary cytokine for fully differentiated and committed T_H_ cells may have over-simplified the complexity and flexibility of T cells. The differences noted between *in vitro* and *in vivo* systems in this review emphasize the importance of understanding the limitations of experimental systems. New and improved technical approaches will be essential in future research, especially with regard to identifying mechanisms of plasticity. It is, as yet, unclear which mechanisms contribute to plasticity and whether there are common triggers of plasticity among experimental systems or even between subsets. Undoubtedly, further research in this area will help us comprehend not just the extreme capabilities of the immune system but how the immune response functions best and how this can be harnessed.

## 10. Acknowledgements

S.M.C., V.S.P. and M.S.W. are funded by the MRC (MRC File Reference number MC_UP_A253_1028) and a Lady TATA foundation grant awarded to M.S.W. We would also like to thank Isobel Okoye, Yashaswini Kannan and Stephanie Czieso for helpful discussions. We apologize to our many colleagues whose important work we did not mention in this review due to space limitations.
